# Molecular Functions and Physiological Roles of Gustatory Receptors of the Silkworm *Bombyx mori*

**DOI:** 10.3390/ijms251810157

**Published:** 2024-09-21

**Authors:** Ryoichi Sato

**Affiliations:** Graduate School of Bio-Application and Systems Engineering, Tokyo University of Agriculture and Technology, Naka 2-24-16, Koganei 184-8588, Tokyo, Japan; ryoichi@cc.tuat.ac.jp

**Keywords:** insect, silkworm, *Bombyx mori*, gustatory receptor, taste receptor, plant secondary metabolites, phylogenetic tree, maxyllary palp, enteroendocrine cell, midgut

## Abstract

Complete elucidation of members of the gustatory receptor (Gr) family in lepidopteran insects began in the silkworm *Bombyx mori*. Grs of lepidopteran insects were initially classified into four subfamilies based on the results of phylogenetic studies and analyses of a few ligands. However, with further ligand analysis, it has become clear that plant secondary metabolites are important targets not only for Grs in the bitter subfamily but also for the *Drosophila melanogaster* Gr43a orthologue subfamily and Grs in the sugar subfamily. Gene knockout experiments showed that *B. mori* Gr6 (BmGr6) and BmGr9 are involved in the recognition of the feeding-promoting compounds chlorogenic acid and isoquercetin in mulberry leaves by the maxillary palps, suggesting that these Grs are responsible for palpation-dependent host recognition without biting. On the other hand, BmGr expression was also confirmed in nonsensory organs. Midgut enteroendocrine cells that produce specific neuropeptides were shown to express specific BmGrs, suggesting that BmGrs are involved in the induction of endocrine secretion in response to changes in the midgut contents. Furthermore, gene knockout experiments indicated that BmGr6 is indeed involved in the secretion of myosuppressin. On the other hand, BmGr9 was shown to induce signal transduction that is not derived from the intracellular signaling cascade mediated by G proteins but from the fructose-regulated cation channel of BmGr9 itself. Cryogenic electron microscopy revealed the mechanism by which the ion channel of the BmGr9 homotetramer opens upon binding of fructose to the ligand-binding pocket. Research on BmGrs has contributed greatly to our understanding of the functions and roles of Grs in insects.

## 1. Introduction

Herbivorous monophagous or oligophagous insects only eat certain plants, while polyphagous insects eat a variety of plants. This is due to the physiological systems that have evolved in response to the allelochemicals produced by host plants and the corresponding recognition systems that developed in herbivorous insects [1]. Therefore, understanding the recognition systems is an important issue in entomology that is essential for pest management. Taste plays a major role in insect–host plant recognition [2,3,4]. Therefore, to understand the mechanisms of host plant recognition, it is necessary to understand the functions and roles of the set of taste receptors of a given insect species.

A great deal of progress was made in understanding the chemical ecology of egg laying in the adult swallowtail butterfly *Papilio xuthus* in the 1980s. Secondary plant metabolites were shown to be received by taste neurons in the tarsi of the legs and used as the main indicators for host plant recognition [3,5]. There has been a great deal of research on the chemical ecology of food recognition and feeding of larvae in the silkworm *Bombyx mori* for the promotion of sericulture in Japan, and the results have been published in domestic and international journals. Understanding of the mechanisms of host recognition and feeding induction in lepidopteran insects began with chemical ecological analysis of mulberry leaf recognition and feeding induction mechanisms in silkworm larvae. These studies showed that secondary metabolites present in mulberry leaves, chlorogenic acid (CGA), isoquercetin (ISQ), and β-sitosterol (βsito) have feeding-promoting activity and play roles in the recognition of mulberry leaves [6,7,8].

An understanding of the neurons in which organs specifically perceive each taste compound can provide insight into the host recognition system. Electrophysiological analysis of the maxillary galea of silkworm larvae revealed that neurons in the lateral styloconic sensilla respond to sugars, while neurons in the central styloconic sensilla respond to repellents [9,10]. In addition, the presence of neurons capable of responding to inositol and repellents was confirmed in the epipharyngeal sensilla on the labrum [11]. However, it remained unclear which organs had neurons responsible for the recognition of CGA, ISQ, and βsito. On the other hand, neurons responding to glucosinolates, a host marker, were identified in both the lateral and central styloconic sensilla of the cabbage white butterfly *Pieris rapae* [12]. Therefore, there was no consistent unified view regarding which organ is responsible for host recognition in lepidopteran insects.

The first insect in which taste receptors were identified was the fruit fly *Drosophila melanogaster*. The trehalose receptor was identified first [13], followed by the determination of all gustatory receptors (Grs) [14]. Complete elucidation of Grs in lepidopteran insects began with the silkworm [15,16,17] and has since been expanded to several insects, including the monarch butterfly *Danaus plexippus*, postman butterfly *Heliconius melpomene*, cotton bollworm *Helicoverpa armigera*, painted lady butterfly *Vanessa cardui*, and diamondback moth *Plutella xylostella* [18,19,20,21,22,23]. Phylogenetic analysis showed that *B. mori* Grs (BmGrs) consists of four subfamilies: the sugar subfamily, which includes BmGr4, BmGr5, BmGr6, BmGr7, and BmGr8; the D. melanogaster GR 43a (DmGr43a) orthologue subfamily, which includes BmGr9 and BmGr10; the carbon dioxide (CO_2_) subfamily, which includes BmGr1, BmGr2, and BmGr3; and the bitter subfamily, which includes the remaining BmGrs [15,16,17]. This subfamily structure has also been shown to be applicable to other lepidopteran insects [18,19,20,21,22,23]. As the ligands of BmGr and other insect Grs in each subfamily have been identified, it has become clear that the original subfamily names are not necessarily appropriate [12,24,25,26,27]. In addition, analysis of tissue expression profiles has suggested that insect Grs are expressed not only in the sensory organs but also in various other organs, including the midgut and brain, and are therefore not simply associated with taste reception [28,29,30,31].

Research on insect Grs is now beginning to provide insights not only into taste reception but also with a view to gaining a new understanding of insect physiology and behavior. Gene knockout experiments have shown that BmGrs function not only in silkworm host recognition by taste but also play an important role in controlling neuropeptide secretion in the midgut [26,32]. Furthermore, analyses of BmGr showed that insect Grs are ion-channel-type receptors, similar to insect olfactory receptors (Ors) [33,34], and the construction of a tertiary structure model based on the results of cryogenic electron microscopy (cryo-EM) contributed to the elucidation of the channel opening mechanism of insect taste receptors that depends on tastant reception [35,36]. This review introduces the results of research on the functions and roles of BmGrs published following the elucidation of all members of the BmGr family.

## 2. Structure- and Ligand-Specific Ion Channel Opening Function of BmGrs

In a heterologous BmGr9 expression system in human HEK293T cells, neither the inhibitor of phospholipase C, U73122, nor the non-hydrolyzable form of GDP, GDP-βS, suppressed Ca^2+^ influx, while chelation of extracellular Ca^2+^ with EGTA showed a suppressive effect on Ca^2+^ influx. These results suggested that the BmGr9-dependent ion channel activity originates from the fructose-regulated cation channel BmGr9 itself [33]. Similarly, BmGr10 was also suggested to form cation channels in a heterologous expression system in *Xenopus laevis* oocytes and HEK293T cells [34]. These findings suggested that insect Gr is an ionotropic channel regulated by taste substances. In a system where Gr was expressed in *Drosophila* neurons, the expression of a single molecule alone did not result in any response to sugar, CO_2_, or bitter compounds, indicating the necessity of intracellular coexpression of other Grs [37,38,39]. Similarly, functional expression of the CO_2_ receptor required coexpression of AaGr1 and AaGr3 in *Aedes aegypti* and HarmGr1 and HarmGr3 in *H. armigera* [40,41]. These observations suggested that many insect Grs require coexpression of other Grs, just as DmOr83b is required for DmOrs in *D. melanogaster* [42,43]. However, both BmGr9 and BmGr10 showed cation channel function when expressed as a single species in a heterologous expression system [33,34], and most lepidopteran Grs also showed channel function when expressed as a single species of Gr. That is, insect Grs may function as heteromeric complexes depending on the insect group or the type of ligand, but most Grs in lepidopteran insects can function as homomeric complexes.

**Figure 1:**
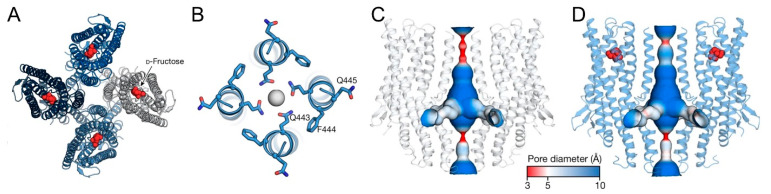
A model for the structure and gating of BmGr9 indicated in [36]. (**A**) Ribbon model of BmGr9 bound to D-fructose (red) shown from the top. (**B**) Close-up views of the pore helices in the presence of D-fructose shown from the top. (**C**,**D**) The ion permeation pathway of BmGr9, colored according to pore diameter, in the absence (**C**) or presence (**D**) of D-fructose (red spheres).

BmGr9 was shown to function as a monomeric complex and act as a cation channel in a model developed based on the results of cryo-EM analysis [35,36]. In this model, the channel molecule is composed of a homotetramer of BmGr9 (Figure 1A,B), and each subunit is composed of seven transmembrane domains (Figure 1C,D), designated as S1 to S7, with S7 divided into S7a and S7b. The central pore is mainly surrounded by S7b helices. In the transmembrane region, S2 to S6 form a ligand-binding pocket extending from the extracellular surface to almost halfway through the membrane (Figure 1D). All Gr proteins share a conserved motif (TYhhhhhQF, h = any hydrophobic residue) in S7b. When fructose binds to the pocket, phenylalanine (F) residues in this motif disappear from the pore surface, while glutamine (Q) residues appear (Figure 1A,B), increasing the affinity for cations and inducing ion channel function [35,36]. The binding of sugars to the ligand-binding pocket of BmGr9 was simulated, and fructose was shown to bind to multiple sites via hydrogen and hydrophobic bonds, resulting in a conformational change in the S7b helices. On the other hand, L-sorbose was also bound but was unable to induce such a conformational change due to differences in the binding sites [36]. In a two-electrode voltage clamp experiment using *Xenopus* oocytes, D-fructose showed channel opening activity for BmGr9. On the other hand, L-sorbose, D-glucose, and D-galactose did not show channel-opening activity but did inhibit the channel-opening activity induced by D-fructose [33]. In another calcium imaging study using BmGr9-transfected HEK293T cells and the calcium indicator Fluo-4 AM, BmGr9 opened the channel in response to 1 aM D-fructose [26], but did not open the channel until the concentration of D-glucose was 1 fM or higher, that of myo-inositol was 10 pM or higher, or that of sucrose was 1 μM or higher. These results suggested that a variety of sugars can bind to the pocket of BmGr9, but how they bind to the potential binding sites in the pocket is important for efficiently altering the conformation of S7b [36].

## 3. Subfamilies Comprising the BmGr Phylogenetic Tree and Ligands of BmGrs

**Figure 2:**
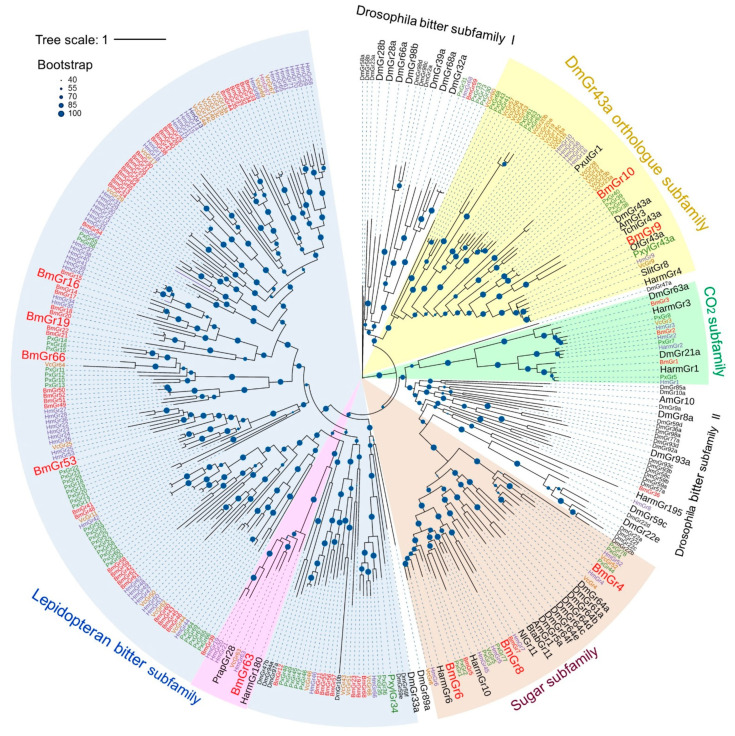
Phylogenetic relationships of Grs from four lepidopteran insects and the fruit fly, and several insect Grs with identified ligands. The amino acid sequences of the total set of Grs of the silkworm *B. mori* (BmGr) [17] (red), painted lady butterfly *V. cardui* (VcGr) [21] (orange), postman butterfly *H. melpomene* (HmGr) [19] (blue), diamondback moth *P. xylostella* (PxGr) [22] (green), and fruit fly *D. melanogaster* (DmGr) (FlyBase: http://flybase.org/reports/FBgg0000079.htm, accessed on 1 September 2024) (black) and Grs (black) for which ligands were identified—HarmGr4, 6, 10, 180, and 195 from the cotton bollworm *H. armigera* [20], OfGr43a from the European corn borer *Ostrinia furanacalis* [44], SlGr8 from the tobacco cutworm *Spodoptera litura* [45], TchiGr43a from the egg parasitoid *Trichogramma chilonis* [46], NlGr11 from the brown planthopper *Nilaparvata lugens* [47], PxutGr1 from the swallowtail butterfly *P. xuthus* [48], and AmGr1, 3, and 10 from the honeybee *Apis mellifera* [49] were aligned with MAFFT v.7 (https://mafft.cbrc.jp/alignment/server/, accessed on 1 September 2024). The IQ-TREE web server (https://iqtree.cibiv.univie.ac.at/, accessed on 1 September 2024) [50] generated the maximum-likelihood tree using the JTT + F + I + G4 model. Bootstrap analysis was carried out with 1000 replicates. The phylogenetic tree was visualized using iTOL (https://itol.embl.de/, accessed on 1 September 2024) [51]. The results of bootstrap analysis are shown as percentages by the size of the blue dots. Putative subfamilies with partial ligand information are shown in brown (sugar), yellow (DmGr43a orthologue), green (carbon dioxide), and blue (lepidopteran bitter). Certain characteristic lineage in lepidopteran bitter subfamily is shown in pink. Large letters indicate Grs for which ligand analysis has progressed.

The phylogenetic relations of Grs have been analyzed in several lepidopteran insect species in addition to *D. melanogaster* [14], including *B. mori* [17], *H. melpomene* [19], *V. cardui* [21], and *P. xylostella* [22]. Taking into account the types of ligands and the results of the analysis of tandem gene clusters on chromosomes, the lepidopteran Grs have been putatively classified into four subfamilies: the DmGr43a orthologue subfamily, the sugar subfamily, the CO_2_ subfamily, and the bitter subfamily (Figure 2). The bitter receptor subfamily members of most lepidopteran insects were shown to form clades distinct from the members of the *Drosophila* bitter subfamily [18,21] (Figure 2).

**Figure 3:**
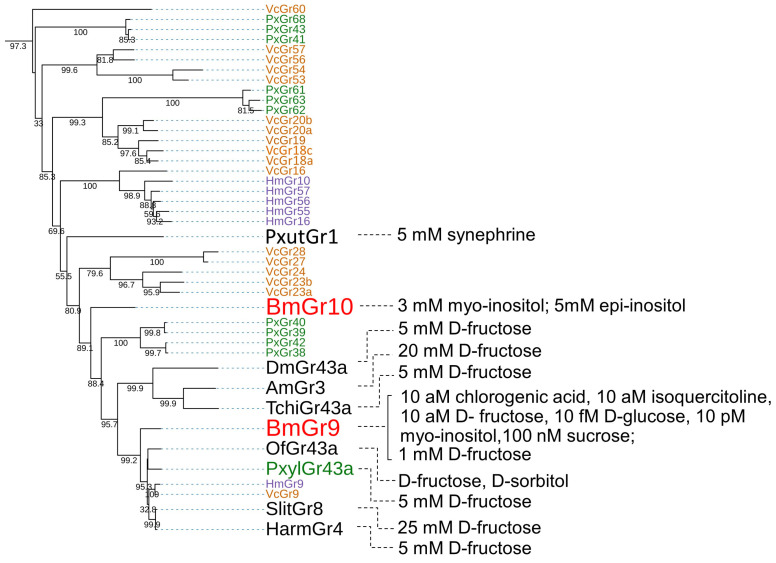
Phylogenetic tree of DmGr43a orthologue subfamily members and their reported ligands. The phylogenetic tree was drawn in the same way as in Figure 2 except that it was drawn in rectangular mode by iTOL (https://itol.embl.de/, accessed on 1 September 2024) [51]. Grs from *B. mori, V. cardui, H. melpomene, P. xylostella* and other insects were respectively written in red, orange, blue, green, and black. Ligands of Grs were cited as follows: PxutGr1, [48]; BmGr10, [34]; DmGr43, [52]; AmGr3, [53,54]; TchiGr43a, [46]; BmGr9, [26,33]; OfGr43a, [44]; PxylGr43a, [55]; SlitGr8, [45]; HarmGr4, [56,57].

### 3.1. DmGr43a Orthologue Subfamily

#### 3.1.1. Ligands of BmGr9

Gene knockout experiments showed that neurons in the maxillary palp are involved in the response to the feeding stimulant CGA at 10 aM and to ISQ at 1 aM in silkworm larvae [26] (Figure 3). On the other hand, experiments using a calcium imaging system in HEK293T cells showed that BmGr9 responded to both CGA and ISQ at the 10 aM level, and this level was closely correlated with the response sensitivity of neurons in the maxillary palp [26]. As described later, the repellent neurons of the maxillary palp can detect coumarin at 1 aM [58]. BmGr53 is expressed in the maxillary palp, and in the same calcium imaging system in HEK293T cells, the EC50 of coumarin to BmGr53 was shown to be 58.18 zM. This made BmGr53 a receptor candidate that can be used to explain the sensitivity of the maxillary palp for detection of coumarin [59]. In contrast, BmGr4 was shown to respond to D-sucrose and D-glucose at 10 mM in the same calcium imaging system in HEK293T cells [30]. The concentrations of these sugars were consistent with those of sugars in the diet. Therefore, it is possible that the high sensitivity shown by HEK293T cells is correlated with the in vivo sensitivity. The EC50 of D-fructose for BmGr9 was approximately 10 aM in this system [26], but 56 mM and 35 mM, respectively, in heterologous expression systems consisting of *Xenopus* oocytes and HEK293T cells in another laboratory [33]. The reason for the difference in response sensitivity of BmGr9 between the two laboratories is unclear and requires further research.

#### 3.1.2. Ligands of BmGr10

In heterologous expression in *Xenopus* oocytes and HEK293T cells, it was shown that BmGr10 responds to the sugar alcohols myo-inositol and epi-inositol but not to glucose, fructose, galactose, sucrose, raffinose, trehalose, maltose, mannitol, or sorbitol [34] (Figure 3). In the *Xenopus* oocyte system, the EC50 values of myo-inositol and epi-inositol for BmGr10 were 44.9 mM and 27.6 mM, respectively [34]. On the other hand, experiments using a calcium imaging system with HEK293T cells and Fluo-4 AM showed that BmGr10 did not respond to 1 nM coumarin, 1 nM caffeine, or 1 nM pilocarpine, but did respond to 1 nM myo-inositol [59]. These findings suggest that BmGr10 is a receptor with high specificity for the two inositol stereoisomers.

#### 3.1.3. DmGr43a Orthologue Subfamily Members of the Lepidopteran Insects

As mentioned above, BmGr9 is closely related to DmGr43a (Figure 2 and Figure 3) and was highly selective for fructose [26,33] (Figure 2). In addition, it has been reported that the ligands of DmGr43a and its orthologues, AmGr3 in *Apis mellonella*, SlGr8 in *Spodoptera litura*, and PxGr43a in *P. xylostella*, are selective for fructose [45,52,53,54,55] (Figure 3). HaGr4 of *H. armigera* was also reported to be specific for fructose [56], but a molecule named HaGr9, which seemed to be the same as HaGr4, responded to D-maltose and D-galactose in addition to fructose [57]. *Ostrinia furnacalis* Gr43 has been reported to be involved in the reception of D-fructose and D-sorbitol [44]. Based on these findings, fructose appears to be an important ligand for a Gr lineage that includes DmGr43a and BmGr9. On the other hand, DmGr43a acts as an internal nutrient sensor in the brain, monitoring hemolymph fructose levels to control satiety [52]. Therefore, it is possible that the DmGr43a orthologue is conserved in many insects because it fulfills such a physiological function.

The ligands of BmGr9 included not only D-fructose but also CGA and ISQ [26], which are known to be feeding stimulants for silkworm larvae [7,8]. In addition, myo-inositol is the ligand for BmGr10 [34], and its isomer chiro-inositol is an oviposition inducer for *P. xuthus* [2,60]. In addition, sequoyitol, a methyl ether of myo-inositol, is an oviposition stimulant for the Aristolochiaceae-feeding swallowtail *Atrophaneura alcinous* [61]. Therefore, BmGr9 and BmGr10 have characteristics of receptors for plant secondary metabolites as host markers. PxutGr1-expressing Sf9 cells responded to synephrine and octopamine [48] (Figure 2 and Figure 3). Synephrine is a secondary metabolite of citrus and is one of the egg laying inducers for adult *P. xuthus* [2,60]. Its receptor, PxutGr1, appears to be located in an expanded lineage outside of BmGr9 and BmGr10 [21] (Figure 2 and Figure 3).

In some lepidopteran insects, species-specific gene duplications associated with host expansion have been reported in the Grs. Such duplication molecules are found in the DmGr43a orthologue subfamily, with 22 in *V. cardui*, 14 in *Vanessa indica*, 16 in *Araschnia burejana*, 14 in *H. melpomene*, and 17 in *P. xylostella* [19,21,22]. These Grs have been speculated to be involved in the recognition of plant secondary metabolites that act as host markers (Figure 3). Taken together, these findings suggest that the DmGr43a orthologue subfamily contains many plant secondary metabolite receptors involved in host recognition. Ten types of secondary metabolites have been reported that are citrus markers and egg laying attractants for *P. xuthus* [2,60]. Therefore, receptors for citrus markers other than PxutGr1 are expected to be found in *P. xuthus* as members of the DmGr43a orthologue subfamily in future studies.

### 3.2. Sugar Subfamily

#### 3.2.1. Ligands of BmGr4

**Figure 4:**
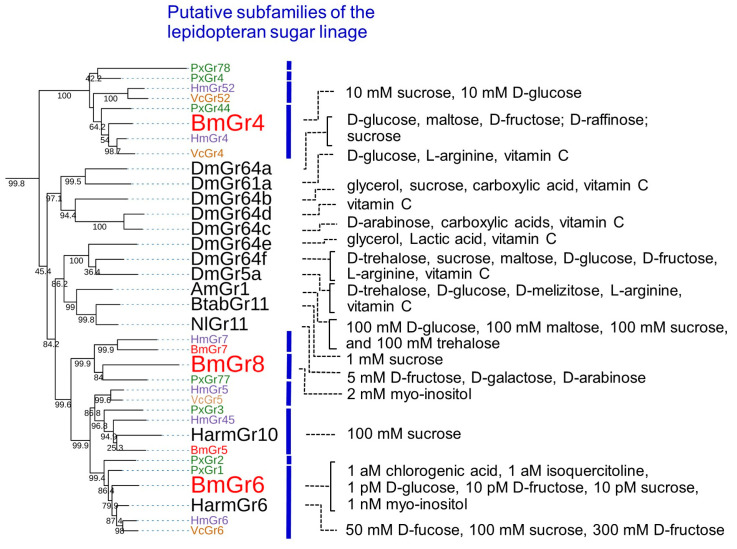
Phylogenetic tree of sugar subfamily members and their reported ligands. The phylogenetic tree was drawn in the same way as in Figure 2 except that it was drawn in rectangular mode by iTOL (https://itol.embl.de/, accessed on 1 September 2024) [51]. Grs from *B. mori, V. cardui, H. melpomene, P. xylostella* and other insects were respectively labeled red, orange, blue, green, and black. Ligands of Grs were cited as follows: BmGr4, [30]; HarmGr10, [62]; DmGr64a, [63,64,65]; DmGr61a, [27,66,67]; DmGr64b, [27,65]; DmGr64d, [27]; DmGr64c [27,65]; DmGr64e [27,65]; DmGr64f, [27,38,67]; DmGr5a, [13,27,64,65,67]; AmGr1, [68]. BtabGr11, [69]; NlGr11, [47]; BmGr8, [16]; BmGr6, [26]; HarmGr6, [62].

In studies using a calcium imaging system with Fluo-4 AM in HEK293T cells, BmGr4 was shown to respond to 10 mM D-sucrose and D-glucose [30] (Figure 4). However, no response was observed to 10 mM D-mannose, trehalose, D-raffinose, D-sorbitol, myo-inositol, ISQ, and CGA, or 50 mM leucine and isoleucine.

#### 3.2.2. Ligands of BmGr6

Calcium imaging experiments in HEK293T cells using Fluo-4 AM showed that BmGr6 has a high sensitivity to CGA and ISQ at the 1 aM level. On the other hand, BmGr6 also responded to sugars, such as D-glucose at 1 pM, D-fructose and sucrose at 10 pM, and myo-inositol at 1 nM [26].

#### 3.2.3. Ligands of BmGr8

Experiments using the calcium imaging system in Sf9 cells showed that BmGr8 responded to 2 mM myo-inositol. However, it did not respond to other sugars, such as D-sucrose, D-maltose, D-trehalose, epi-inositol, allo-inositol, and scyllo-inositol [16]. Unlike hexose, myo-inositol is a cyclohexane polyol.

#### 3.2.4. Sugar Subfamily Members of the Lepidopteran Insects

Of the Gr groups used in the construction of this phylogenetic tree, those that are thought to include almost all members are BmGrs (from *B. mori*), VcGrs (from *V. cardui*), HmGrs (from *H. melpomene*), and PxGrs (from *P. xylostella*). For BmGrs, VcGrs, HmGrs, and PxGrs, all Grs members were used in constructing the phylogenetic tree (Figure 2). However, orthologues in the same lineage in the sugar subfamily are not necessarily present in these four insect species (i.e., *B. mori*, *V. cardui*, *H. melpomene*, and *P. xylostella*) (Figure 4). Specifically, the conservation of Grs among the other three insect species in the lineages of BmGr4, BmGr5, and BmGr6 is high, but the conservation is low in the lineages of BmGr7 and BmGr8 (Figure 4). In addition, four lineages did not include any BmGrs (Figure 4). These observations suggest that species- or group-specific expansion or loss of Grs has occurred, as was seen in the DmGr43a orthologue subfamily (Figure 3), which suggests that the types of sugars used to identify food are not necessarily common among insects [21].

HaGr6, which is thought to be in a lineage with no orthologue to BmGr, responded to D-fucose, D-sucrose, and D-fructose [62] (Figure 4). HaGr10, which is in the lineage of BmGr5, responded to D-sucrose [62] (Figure 4). Therefore, the ligand specificities of Grs from different lineages were somewhat similar. Dmgr5a, DmGr61a, DmGr64a, DmGr64b, DmGr64c, DmGr64e, and DmGr64f have been shown to be involved in the recognition of different sugars [13,38,52,63,64,65,66] (Figure 4). NlGr11 from the brown planthopper *N. lugens*, which is thought to belong to the lineage of Dmgr5a, DmGr64e, and DmGr64f, responded to fructose, galactose, and arabinose [47] (Figure 4). AmGr1 from *A. mellifera*, which is also thought to belong to the lineage of these DmGrs, responded to glucose, maltose, trehalose, and sucrose [67] (Figure 4). In addition, BtabGr11 from the whitefly *Bemisia tabaci* responded to D-sucrose [68] (Figure 4). Therefore, Grs that are thought to be in the same lineage recognize several types of sugars simultaneously, and there is no strict conservation of the sugars recognized (Figure 4). The expansion and contraction observed within the sugar subfamily members of a single insect species is therefore thought to be the result of each insect responding to differences in the sugars available when altering its host range by changing the arrangement of Grs with slightly different specificities and recognition spectra (Figure 4).

DmGr5a, DmGr61a, and DmGr64f have been shown to be involved in the detection of L-arginine [24]. Therefore, some of the Grs in the sugar subfamily, including the five BmGrs of the silkworm, may also accept any amino acid. In fact, responses to L-isoleucine and L-leucine have been reported in an unidentified neuron in the lateral sensilla of the maxillary galea in the silkworm ([69] in Japanese with English summary). In addition, the presence of amino acid neurons that contribute to the induction of proboscis extension reflex has been reported in *H. armigera* adults [70].

As mentioned above, BmGr6 is highly sensitive to CGA, which are esters of caffeic acid and quinic acid, and ISQ, a flavonoid [26]. BmGr8 also responds to the cyclohexane polyols myo-inositol and epi-inositol [16]. In Drosophila, DmGr5a, DmGr61a, DmGr64b, DmGr64c, DmGr64e, and DmGr64f, along with two ionotropic receptors, IR25a and IR76b, have been shown to be involved in the perception of vitamin C, a secondary metabolite with an unsaturated lactone ring [27] (Figure 4). Based on these findings, the sugar subfamily receptors appear to be receptors not only for hexose and its dimer but also for cyclohexane polyol and secondary plant metabolites. Previous studies of the ligands of the sugar subfamily were influenced by existing knowledge of the Gr subfamily, and the ligands of the sugar subfamily were assumed to be sugars. Further studies are expected to show that many Grs of the sugar subfamily also respond to secondary metabolites.

### 3.3. Bitter Subfamily

#### 3.3.1. Ligands of BmGr16, BmGr19, and BmGr53

**Figure 5:**
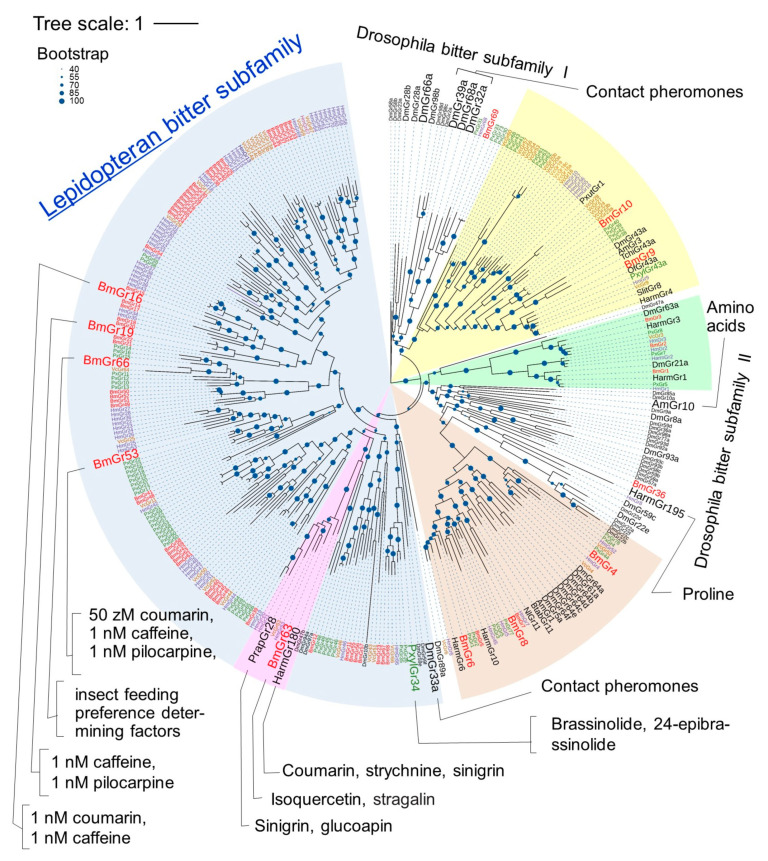
Phylogenetic tree of lepidopteran bitter subfamily members and their reported ligands. The phylogenetic tree was drawn in the same way as in Figure 2. Grs from *B. mori*, *V. cardui*, *H. melpomene*, *P. xylostella* and other insects were respectively written in red, orange, blue, green, and black. Ligands of Grs were cited as follows: BmGr16, [59]; BmGr19, [59]; BmGr66, [71]; PrapGr28, [12]; BmGr63, [25]; HarmGr180, [72]; PxylGr34, [22]; AmGr10, [73]; DmGr32a, 33a, 39a, and 68a, [74,75,76]; HarmGr195, [20].

Calcium imaging experiments with Fluo-4 AM in HEK293T cells showed that BmGr16 responded to coumarin and caffeine at 1 nM, but not to 1 nM pilocarpine, nicotine, inositol, sucrose, or ISQ [59]. BmGr19, previously reported as BmGr18 [58] (Figure 5), responded to 1 nM coumarin, caffeine, and pilocarpine, but not to 1 nM nicotine, inositol, sucrose, or the flavonoid ISQ [59] (Figure 5). BmGr53 responded to 1 nM caffeine and pilocarpine, but not to 1 nM nicotine, inositol, sucrose, or ISQ [59] (Figure 5). Therefore, despite their distant phylogenetic relations, Gr16, Gr19, and Gr53 responded to partially overlapping plant secondary metabolites with dissimilar structures [59]. The EC50 of coumarin for BmGr53 was 58.18 zM [59]. Coumarin was shown to indeed be a feeding repellent, as it inhibited feeding on sucrose-containing agar in silkworm larvae, and the EC50 of coumarin for neurons in the maxillary palp of larvae was approximately 1 aM [58]. Therefore, the sensitivity of the coumarin-responsive neurons in the maxillary palp and the sensitivity of BmGr53 to coumarin appeared to be correlated. However, as mentioned in Section 3.1.1, the significance of this correlation requires further study.

#### 3.3.2. Ligands of Bmgr63

In experiments using a voltage clamp recording in *Xenopus* oocytes, Bmgr63 was shown to respond to the flavonoid glycoside ISQ at 100 µM [25] and weakly to its structural analog astragalin. However, it did not respond to sugars or bitter compounds. In larvae in which expression of BmGr63 in the mouthparts was reduced by 50%, by double-stranded RNA interference (dsRNAi), food intake and growth on an artificial diet supplemented with ISQ were reduced [25]. As ISQ is a feeding stimulant in silkworm larvae [7], it is unlikely that a substance perceived as a feeding stimulant through one receptor would be perceived as a repellent through another receptor. Therefore, although BmGr63 is located in the lepidopteran bitter subfamily, it is likely that it functions as a receptor for feeding stimulants.

#### 3.3.3. Ligands of BmGr66

Silkworm larvae in which BmGr66 was genetically ablated via Cas9/sgRNA-mediated targeted mutagenesis (CRISPR/Cas9) ate leaves of Mongolian oak (Quercus mongolica Fisch. ex Ledeb.), which they do not normally eat [71]. One possible mechanism for this phenomenon is that BmGr66 may respond to repellents. However, BmGr66 depletion did not change the electrophysiological response of the medial styloconic sensilla of the maxillary galea to caffeine and salicin [71]. In addition, as mentioned above, not only the maxillary galea but also the maxillary palps are involved in repellence [58]. Experiments using real-time quantitative reverse transcription PCR (qRT-PCR) showed that BmGr66 was expressed in the labrum and labium, but was expressed at higher levels in the maxilla [71]. Furthermore, a different study showed that BmGr66 was expressed in both maxillary galea and maxillary palps [17]. The possibility that Gr66 responds to the as-yet-unknown repellent substance in the leaves of Mongolian oak cannot be excluded. Although BmGr66 is a member of the lepidopteran bitter subfamily, it is unclear whether it actually responds to repellents.

BmGr66-depleted larvae gained weight while eating fruits of apple (*Malus domestica*) and pear (*Pyrus* spp.) and seeds of soybean (*Glycine max*) and corn (*Zea mays*) [71]. In addition, in a two-choice feeding assay using artificial foods with or without mulberries, they lost their preference for mulberries [71]. These observations suggest that BmGr66-depleted larvae do not require the feeding stimulants contained in mulberries. However, the reason why BmGr66 depletion eliminated the need for feeding stimulants has not been determined, and further research is needed to understand the mechanism by which Bmgr66 depletion removes food preference.

#### 3.3.4. Bitter Subfamily Members of Lepidopteran Insects

In the phylogenetic tree shown in Figure 2, most of the Gr members, except the DmGr43a orthologue subfamily, the sugar subfamily, and the CO_2_ subfamily, constitute a subfamily separate from the *Drosophila* bitter subfamily, as reported previously [18] (Figure 2). Clusters that appear to be formed by species-specific or group-specific gene duplications were found in this subfamily, which has been considered to be the result of adaptation to expansion of or changes in host plants [17,18,19,20,22,77] (Figure 5). Species-specific gene duplication was also observed in the silkworm [17] (Figure 2 and Figure 5). That the expansion of related Grs occurred due to gene duplication is also supported by the observation that highly similar genes are arranged on the same chromosome [17]. In this way, the Grs of the lepidopteran putative bitter subfamily are thought to have formed a group separate from the Drosophila bitter subfamily.

Many DmGrs involved in bitter tastant reception have been identified in the Drosophila bitter subfamily, including DmGr8a, DmGr22e, DmGr28a, DmGr28b, DmGr32a, DmGr33a, Dm39a, DmGr59c, Dmgr66a, DmGr89a, DmGr93a, and DmGr98b [39,78,79,80,81]. Therefore, it is expected that many DmGrs in the *Drosophila* bitter subfamily are indeed bitter receptors. This also suggests that many of the lepidopteran bitter subfamily members are also bitter receptors. In fact, as mentioned above, BmGr16, BmGr19, and BmGr53 in the lepidopteran bitter subfamily have been shown to be bitter tastant (repellent) receptors [58,59] (Figure 5). In addition, PxylGr34 in *P. xylostella* responded to brassinolide (BL) and 24-epibrassinolide, which were found to have feeding-repellent activity in larvae, and knockdown of PxylGr34 expression increased the amount of feeding on BL-treated cabbage leaves [22]. That is, PxylGr34 is considered to be a repellent receptor (Figure 5).

However, it has been reported that this subfamily may contain diverse types of Grs. As mentioned above, BmGr63 may accept ISQ as a feeding stimulant [25]. On the other hand, PrapGr28 of *Pieris rapae*, which is in the same lineage as Bm63, responds to sinigrin (Figure 5). The spike frequencies of female tarsi in response to sinigrin decreased by 30% when PrapGr28 expression was knocked down by 40%, by RNAi [12]. *P. rapae* uses sinigrin as a promoter of larval feeding and adult oviposition [82,83,84], and the lateral sensilla styloconica responds to sinigrin [12]. Therefore, PrapGr28 may be one of the sinigrin receptors that contributes to these functions. On the other hand, Harm180 of *H. armigera* responds to sinigrin but also responds to substances known as insect repellents, such as coumarin and strychnine [72]. Sinigrin is known to be a repellent for many noctuid moths, including *Trichoplusia ni* and *Heliothis virescens* [85,86]. Therefore, sinigrin is thought to play a role as a repellent for *H. armigera*. BmGr63-containing linages may therefore contain a mixture of receptors for feeding stimulants and feeding repellents (Figure 5).

Plant nectars contain as much as 2 mM proline, which is an attractant in some insects [87]. Harm195 in *H. armigera* is expressed in the tarsi of adult *H. armigera* and is thought to be involved in the control of oviposition, and was shown to respond to proline [20]. AmGr10 of *A. mellifera* is expressed in the hypopharyngeal gland and is a receptor that is tuned to amino acids and does not respond to bitter tastes [73] (Figure 5). When AmGr10 is knocked down, worker bee behavior shifts from nursing to foraging, so AmGr10 is thought to be involved in nursing behavior [88]. These Grs were present in the Drosophila bitter subfamily in the phylogenetic tree shown in Figure 5.

The *Drosophila* bitter subfamily includes not only repellent receptors but also receptors with different functions. DmGr32a, Dmgr33a, DmGr39a, and DmGr68a are contact pheromone receptors involved in inducing courtship between males and females in Drosophila [74,75,76]. BmGr36 and BmGr69, as well as some Grs from *P. xylostella* and *H. melpomene*, were also assigned to the bitter subfamily of *Drosophila* in the phylogenetic tree (Figure 5). The ligands of these Grs are of interest. Judging from the bootstrap values, the probabilities that these Grs are members of the bitter subfamily of *Drosophila* are not high, and on repeated analysis, they were often included in the lepidopteran putative bitter subfamily. These Grs with uncertain phylogenetic positions may include those that respond to various ligands other than repellents.

## 4. Gr-Expressing Organs and the Roles of Grs

### 4.1. Spatial Expression of BmGrs

**Figure 6:**
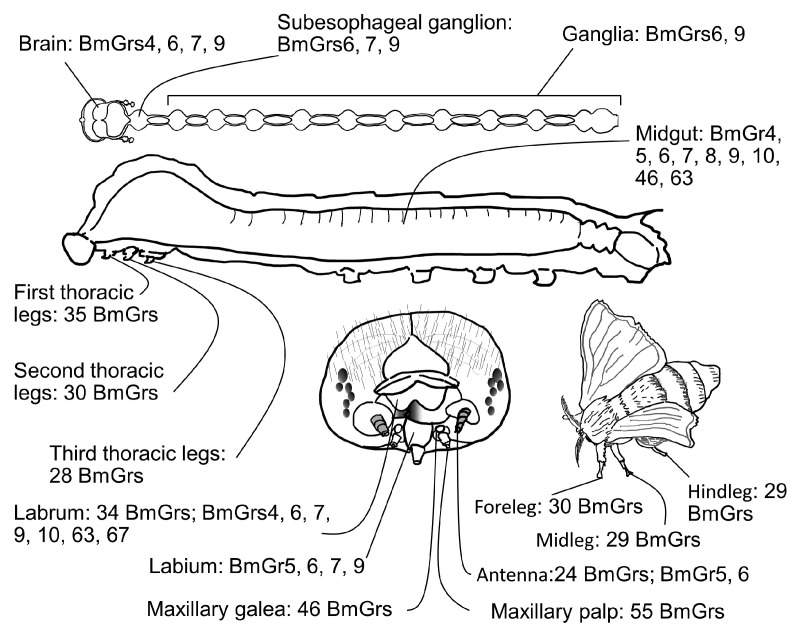
BmGrs whose expression has been reported. The BmGrs expressed in each organ are cited as follows: larval brain, [28,29,30]; larval subesophageal ganglion, [28,29]; larval ganglia, [28,29]; larval midgut, [28,29,30,31,32]; larval first thoracic legs, [17]; larval second thoracic legs, [17]; larval third thoracic legs, [17]; larval labrum, [17,28,29,90]; larval labium, [28,29]; larval maxillary garea, [17]; larval maxillary palp, [17]; larval antenna, [17,28]; adult foreleg, [17]; adult midleg, [17]; adult hindleg, [17].

The chemosensory organs in which BmGrs are expressed were examined by RNA sequencing (RNA-seq) analysis [17]. A total of 46 types of BmGr were expressed in the larval maxillae, 44 types were expressed in the larval thoracic legs, and 52 types were expressed in the adult legs. The expression of such large numbers of BmGrs suggested that these are important taste organs [14]. On the other hand, several Grs showed differences in expression levels between larval and adult stages. For example, BmGr53 and BmGr67 were expressed at higher levels in larvae than in adults, BmGrs27–31 were highly expressed in adult legs, and BmGrs14–17 were only expressed in larval maxillae. BmGrs49–52 were also highly expressed in larvae, but showed very low expression in adults. BmGr2, a CO_2_ receptor candidate, was highly expressed in the antennae of adults, suggesting that CO_2_ detection is important in adults. These observations suggested that some Grs are used for different purposes in adults and larvae or that some have different levels of importance between the two stages [14]. Some Grs showed significant differential expression levels among organs. The inositol receptor BmGr8 [16] was mainly expressed in the maxillary galea, whereas the D-fructose receptor BmGr9 [33] was highly expressed in maxillary palps [14]. In larvae, expression of Bmgr9 was confirmed in the sensory organs, labrum, mandible, maxilla, labium, thoracic leg, and proleg, but not in the antenna [29] (Figure 6). Expression of BmGr6 was also confirmed in chemosensory organs, such as the antenna, labrum, mandible, maxilla, thoracic legs, prolegs, and caudal legs [28] (Figure 6).

Myo-inositol is a feeding-inducing factor in silkworm larvae [7,89]. The labrum of silkworm larvae contains a pair of epipharyngeal sensilla, which contain three GR neurons. One of these, the myo-inositol neuron responded to myo-inositol and was shown to be involved in the induction of feeding [11]. A total of 15 types of BmGr were detected by RT-PCR from three isolated neurons in the epipharyngeal sensillum [90] (Figure 6). Moreover, seven BmGrs—BmGr4, 6, 7, 9, 10, 63, and 67—were identified in an isolated neuron. As BmGr10 responded to myo-inositol [34], this cell was speculated to be the epipharyngeal myo-inositol neuron [90]. However, this neuron did not respond to the sugars D-glucose, sucrose, and D-fructose [26,30], which were shown to be ligands for BmGr4, 6, and 9 [11] (Figure 3 and Figure 4), so there was a discrepancy between expression and response. Although expression was confirmed by RT-PCR, it remains unclear whether the expression level was of biological significance for function. In addition, the responses of epipharyngeal myo-inositol neurons to CGA and ISQ [25,26], which are ligands of BmGr6, BmGr 9, and BmGr 63, have not been investigated [11]. The ligand of BmGr67 is still unknown, but it is in a lineage that is relatively distant from BmGr16, 19, and 53, which have been shown to be receptors for repellents [59] (Figure 5). Therefore, the possibility that BmGr67 is an attractant receptor cannot be excluded. On the other hand, one of the three neurons in the epipharyngeal sensillum has been reported to respond to strychnine nitrate [11]. It is possible that the remaining eight putative deterrent receptors—BmGr15, BmGr16, BmGr17, BmGr21, BmGr35, BmGr53, BmGr56, and BmGr57—are expressed in this single cell, although no attempts have yet been made to isolate this cell.

### 4.2. Roles of BmGr6 and BmGr9 in First Authentication of Food by the Maxillary Palps

**Figure 7:**
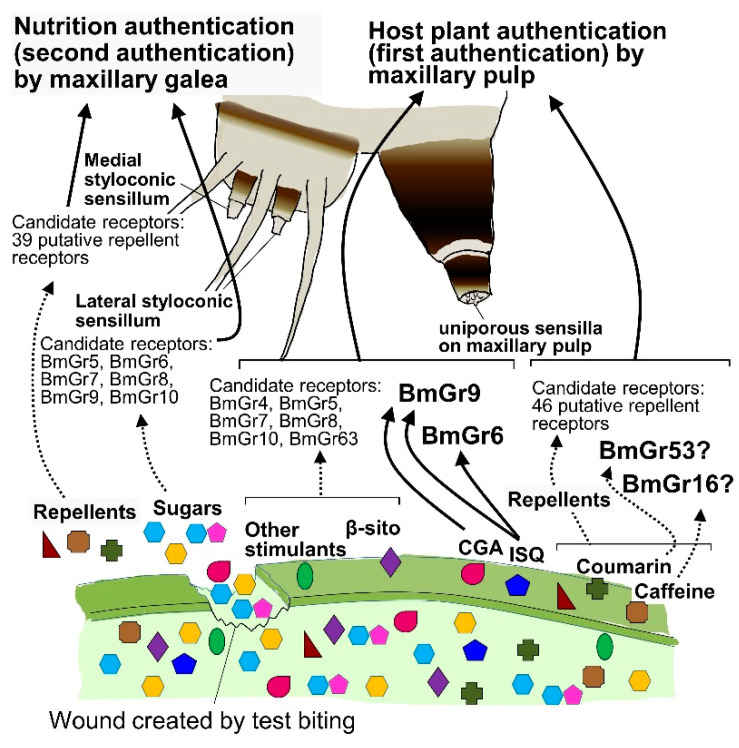
BmGrs that may be involved in the two-factor authentication model of food recognition [8]. Candidate BmGrs were taken from previous reports [17,25,26,59]. CGA, chlorogenic acid; ISQ, isoquercetin; β-sito, β-sitosterol.

As mentioned briefly above, the larvae of the silkworm *B. mori*, which have a well-known dietary preference for mulberry leaves, select and feed on the leaves utilizing a two-factor authentication system [8] (Figure 7). Two sensory organs with different sensitivities work together in this system. The first authentication is performed by palpating the leaf surface with the maxillary palps, where ultrasensitive chemosensory neurons detect mulberry secondary metabolites, including CGA, quercetin glycosides, including ISQ, and the phytosterol, βsito [6,7,8]. The ultrahigh sensitivities—in the 10 aM range for CGA and ISQ and 1 fM range for βsito—are thought to enable the larvae to detect trace amounts of these compounds at the dry leaf surface and identify mulberry leaves [8] (Figure 7). Secondary metabolites found in tissues have been shown to be present in trace amounts on the leaf surface [91]. After this first authentication, the larvae test bite the mulberry leaf and then use the sugars in the tissue fluid that oozes out of the leaf to determine whether it is suitable as food (i.e., second authentication), after which they move on to persistent biting [8] (Figure 7). Here, it is thought that CGA, ISQ, and βsito used in the first authentication act as host markers for the larvae to recognize mulberry leaves. However, these three substances are found in many plants. In fact, silkworm larvae cannot strictly identify mulberry, and not only perform first authentication on Cichorioideae plants, such as *Sonchus oleraceus* and *Taraxacum officinale*, but they also perform second authentication and have a high probability of proceeding to persistent biting [6,58]. It is thought that the larvae are provided a suitable habitat based on the stricter judgment of the adult female, based on odor and taste recognition [92]. These three substances, which are thought to be insufficient for the larvae to identify mulberry among many other plants, may be sufficient for the larvae to distinguish mulberry leaves in such habitats.

The presence of neurons expressing BmGr6 and BmGr9 in the maxillary palp was demonstrated by immunohistochemistry [26]. Furthermore, RNA-seq showed that these two BmGr genes are expressed at high levels in the maxillary palp [17]. As mentioned above, the response sensitivities of BmGr6 and BmGr9 in the maxillary palp and HEK293T cells to CGA and ISQ were similar [26]. Furthermore, the responses of neurons of the uniporous sensillae of the maxillary palps to ISQ observed on electrophysiological recordings were completely abolished in BmGr6 knockout silkworms. On the other hand, BmGr9 knockout reduced the response sensitivity of the maxillary palp to both CGA and ISQ by 1000-fold [26]. Therefore, BmGr6 was shown to play an important role in the recognition of ISQ, while BmGr9 plays an important role in the recognition of CGA and ISQ in the first authentication of food [26] (Figure 7). Furthermore, the knockout of either BmGr6 or BmGr9 reduced the rate of test biting of mulberry leaves by 60% [26]. This limited reduction was convincing enough, as βsito was also shown to be involved in the first authentication of food [8].

### 4.3. Role of Bitter Receptors in the First Authentication of Food by the Maxillary Palps

Silkworm larvae did not eat mulberry leaves coated with the surface material from the leaves of others that they did not eat. Therefore, experiments involving suppression of test biting of leaves that silkworms do not eat showed that repellent reception by palpation makes a major contribution to diet choice [58]. That is, the recognition of many repellents by the maxillary palps is thought to play an important role in repelling silkworms from food other than mulberry leaves (Figure 7), as also reported in *Manduca sexta* [93]. Chip recording analyses showed that neurons in the maxillary palps responded to the repellent coumarin with an EC50 of 1 aM [58]. Therefore, including the recognition of attractants, palpation using the maxillary palps is thought to enable the insects to detect trace amounts of plant secondary metabolites present on the leaf surface and recognize the host plant without ingesting any dangerous substances [8,58].

Candidates for Grs involved in such highly sensitive repellent recognition in the maxillary palps include the putative bitter receptors expressed in these organs. The expression of 46 putative bitter receptors in the maxillary palps was confirmed by RNA-seq [17] (Figure 7). BmGr16, BmGr19, and BmGr53 expression were confirmed in maxillary palps by RT-PCR [59] (Figure 6 and Figure 7). As mentioned in Section 3.3.1, BmGr16, BmGr19, and BmGr53 show very high response sensitivities to repellents [59]. This may enable silkworms to reject plants other than the host, mulberry, without ingesting potentially dangerous substances [58].

### 4.4. Food Recognition through Midgut Expressed BmGrs

#### 4.4.1. Expression and Role of BmGr4 in Midgut Enteroendocrine Cells

**Figure 8:**
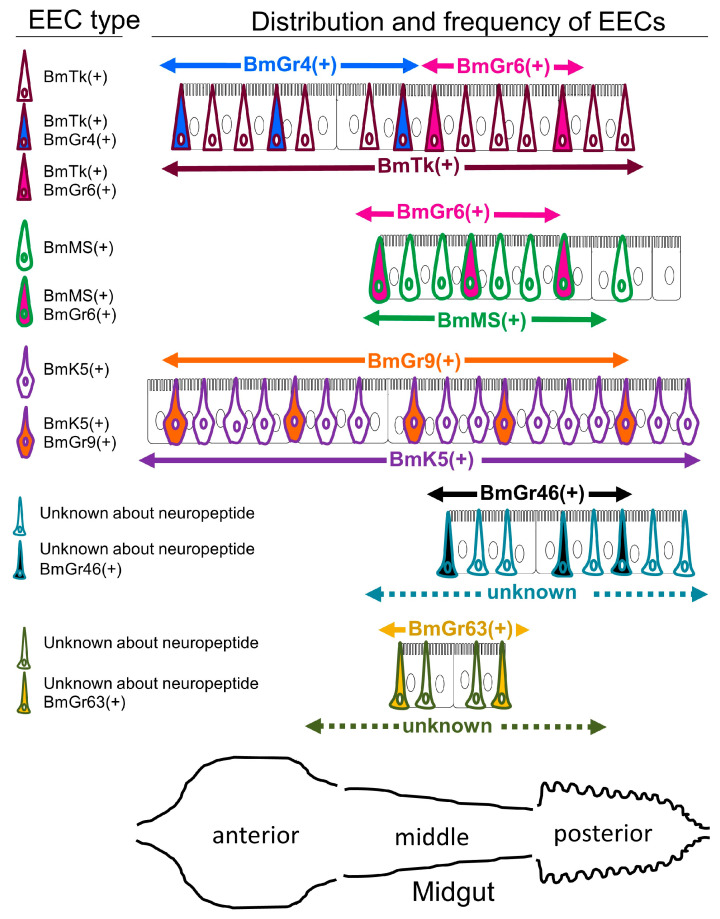
Overview of the expression sites and expression frequencies of BmGrs in midgut EECs. Schematic diagrams of the expression sites and expression frequencies of BmGr4, BmGr6, BmGr9, BmGr46, and BmGr63 were created based on previous reports [28,29,30,31,32]. Arrows indicate regions expressing neuropeptides and BmGrs. BmGr-expressing cells: blue, BmGr4; magenta, BmGr6; orange, BmGr9; black, BmGr46; yellow, BmGr63.

Enteroendocrine cells (EECs) producing tachykinin-related peptide (TRP), which was reported to have feeding-promoting activity and intestinal lipid production-promoting activity [94,95], were found throughout the entire midgut of third instar silkworm larvae [30,31] (Figure 8). These cells were positioned to respond as soon as food arrived in the midgut, within 10 min after the start of feeding [30,31]. In fact, BmTRP secretion occurred as soon as the ingested mulberry leaf reached the anterior midgut [30,31]. BmGr4 was expressed in BmTRP-producing EECs, and the expressing cells were only present in the anterior half of the midgut (Figure 8). That is, they were in a position to sense food as soon as it arrived in the midgut. Experiments in a heterologous expression system in HEK239T cells showed that BmGr4 responds to sucrose and glucose [30]. It was also shown that BmTRP is secreted from the midgut by EECs in response to sucrose and glucose, and that sufficient sucrose and glucose are present in mulberry leaves to induce its secretion [96]. These lines of circumstantial evidence suggest that BmGr4 transmits signals leading to BmTRP secretion in a portion of BmTRP-producing EECs. Direct evidence, such as the results of gene knockout experiments, is urgently required to resolve these issues.

#### 4.4.2. Expression and Role of BmGr6 in Midgut Enteroendocrine Cells

**Figure 9:**
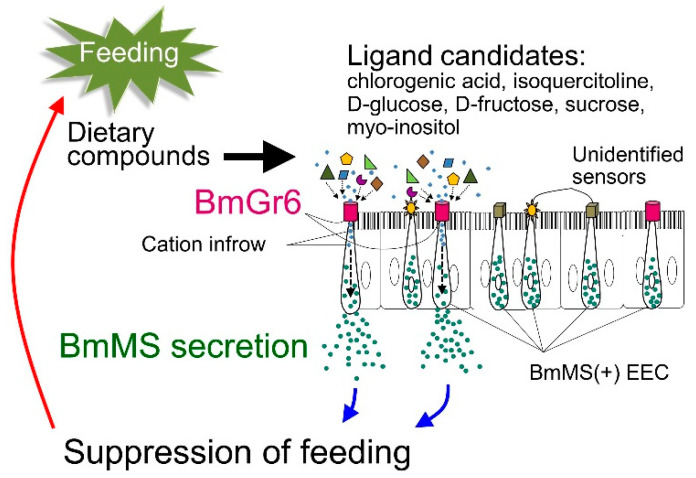
A model for regulation of BmMS secretion in midgut EECs based on the report of [32] on recognition of mulberry leaf components by BmGr6. The epithelial tissue depicted represents the midgut middle region. Ligand candidates for BmGr6 were based on a previous report [26].

BmGr6 was shown to be expressed in a portion of FMRFamide (Phe-Met-Arg-Phe-amide)-producing EECs in the midgut of silkworm larvae [28], and the FMRFamide was shown to be Bommo-myosuppressin (BmMS) [32]. Myosuppressin was reported to be involved in the suppression of food intake in larvae of the African cotton leafworm Spodoptera littoralis and the German cockroach *Blattella germanica* [94,97]. In fifth instar silkworm larvae, BmGr6-expressing BmMS-producing EECs were located in the middle midgut, a position that detects sufficient feeding (Figure 8 and Figure 9). Indeed, the timing of BmMS secretion into the hemocoel was shown to be correlated with the arrival of food at the midgut [31,32]. Furthermore, BmMS secretion from EECs was significantly reduced by BmGr6 knockout. Therefore, there is direct evidence for the involvement of BmGr6 in the induction of BmMS secretion [32] (Figure 9). However, although significant, the inhibition rate of BmMS secretion in BmGr6 knockout experiments was low. This was thought to be because other sensory receptors in addition to BmGr6 are involved in the induction of BmMS secretion from EECs. Indeed, only about 20% of the BmMS-producing EECs in third instar silkworm larvae expressed BmGr6, and it was suggested that other sensors are expressed in the other BmMS-producing EECs [31,32]. The expression of transient receptor potential (TRP) cation channels [98], which are receptors for various stimuli, in EECs has been reported in Drosophila, and the existence of EECs with various other receptors was predicted. As mentioned above, in a heterologous expression system in HEK293T cells, BmGr6 responded to 1 aM CGA and ISQ, 1 pM D-glucose, 10 pM D-fructose and sucrose, and 1 nM myo-inositol [26]. At present, it is unknown which of these substances derived from mulberry leaves is detected by BmGr6 to induce BmMS secretion (Figure 9).

BmGr6 was also expressed in a small proportion of BmTRP-producing cells in the middle to posterior midgut [31,32] (Figure 8). However, the knockout of BmGr6 did not affect the amount of BmTRP secreted after feeding [32]. These observations suggested that BmGr6 does not play a large role in the regulation of BmTRP secretion and that many other sensors are involved in the underlying mechanisms.

#### 4.4.3. Expression of Other BmGrs in Midgut Enteroendocrine Cells

BmGr9 was expressed in approximately 30% of EECs producing K5 (BmK5) in the midgut from the anterior to posterior of silkworm larvae [31] (Figure 8). Five minutes after the start of feeding, the food reached the entrance of the anterior midgut, at which point BmK5 secretion began [31]. Therefore, it was speculated that a portion of BmK5 secretion was mediated by BmGr9. It is unclear what the EECs secrete, but BmGr46 and BmGr63 were also expressed in middle midgut EECs [31] (Figure 8).

The expression of 23 types of neuropeptides in the silkworm midgut was confirmed by the Rapid Amplification of cDNA Ends (RACE) method [99]. On the other hand, reports on the expression of BmGrs in silkworm midgut EECs were limited [28,29,30,31,32]. It is likely that expression of more BmGrs in the midgut EECs will be discovered in future experiments, and that relations between various neuropeptide-producing EECs and BmGr will be clarified.

### 4.5. Roles of BmGrs beyond Taste Recognition

BmGr6 was shown to be expressed in the brain and all ganglia of silkworm larvae [28] (Figure 6). Immunostaining analyses revealed that there were about 10 BmGr6(+) neurons in the brain, and one or two BmGr6(+) neurons in each ganglion. As mentioned above, the blood satiety marker D-fructose can act as a ligand for BmGr6 [52] (Figure 4). However, BmGr6 also shows high sensitivity for secondary plant metabolites, such as CGA and other sugars, such as D-glucose [26] (Figure 4), so the ligand for BmGr6 in these neurons is not yet clear. Neuropil extending from the brain to the corpora cardiaca, corpora alata, and nervus corporis cardiaca 1 and 2, as well as parts of the neuropil extending from the ganglia to the foregut and hindgut, were strongly stained with anti-BmGr6 antibody. In addition, experiments using anti-DmFMRFamide antibodies confirmed that some neurosecretory cells in the brain and ganglia express FMRFamide-related peptides, similar to some EECs [28]. Therefore, BmGr6 may be involved in regulating internal physiological conditions through the secretion of neuropeptides or neurotransmitters.

RT-PCR analyses confirmed the expression of Bmgr9 in the brain and all ganglia of larvae [29] (Figure 6). Immunostaining revealed about eight pairs of BmGr9-expressing cells in the brain and several pairs of BmGr9-expressing cells in most ganglia. The morphology and location of these cells are similar to, but show slight differences from, BmGr6-expressing cells [28]. Indeed, double staining revealed that many BmGr9-expressing cells also expressed BmGr6, while others did not [29]. In *Drosophila*, it has been suggested that DmGr43a-expressing cells in the brain may have a mechanism for satiety-dependent control of feeding mediated by neuropeptide F secretion [52]. On the other hand, most of the BmGr9-expressing cells in the brain were stained with an antibody against *D. melanogaster* neuropeptide F, suggesting that they were neuropeptide F-producing cells [29]. On the other hand, BmGr9 is thought to be an orthologue of fructose-responsive DmGr43a [16,26] (Figure 2 and Figure 3). If multiple neurons in the brain respond to fructose as a marker of satiety, fructose may not only control feeding behavior [52] but may also be involved in the control of a wider variety of behavioral and physiological effects. However, as mentioned above, BmGr9 can respond to a variety of substances with high sensitivity [26] (Figure 3). Therefore, caution is required in concluding whether fructose is the true ligand for all BmGr9-expressing cells in the brain and ganglia.
